# Engineering a surrogate human heteromeric α/β glycine receptor orthosteric site exploiting the structural homology and stability of acetylcholine-binding protein

**DOI:** 10.1107/S205225251901114X

**Published:** 2019-09-04

**Authors:** Alice Dawson, Paul Trumper, Juliana Oliveira de Souza, Holly Parker, Mathew J. Jones, Tim G. Hales, William N. Hunter

**Affiliations:** a Division of Biological Chemistry and Drug Discovery, School of Life Sciences, University of Dundee, Dundee DD1 5EH, Scotland; b Division of Systems Medicine, School of Medicine, Ninewells Hospital, University of Dundee, Dundee DD1 9SY, Scotland

**Keywords:** acetylcholine-binding protein, crystal structures, glycine receptor, ligand-gated ion channel, nicotine, strychnine, tropisetron

## Abstract

Protein-engineering methods have been exploited to produce a surrogate system for the extracellular neurotransmitter-binding site of a heteromeric human ligand-gated ion channel, the glycine receptor. This approach circumvents two major issues: the inherent experimental difficulties in working with a membrane-bound ion channel and the complication that a heteromeric assembly is necessary to create a key, physiologically relevant binding site. Residues that form the orthosteric site in a highly stable ortholog, acetylcholine-binding protein, were selected for substitution. Recombinant proteins were prepared and characterized in stepwise fashion exploiting a range of biophysical techniques, including X-ray crystallography, married to the use of selected chemical probes. The decision making and development of the surrogate, which is termed a glycine-binding protein, are described, and comparisons are provided with wild-type and homomeric systems that establish features of molecular recognition in the binding site and the confidence that the system is suited for use in early-stage drug discovery targeting a heteromeric α/β glycine receptor.

## Abstract 2

The development and characterization of a surrogate system to support drug discovery targeting a complex heteromeric human glycine receptor is described.

## 1. Introduction   

Pentameric ligand-gated ion channels (pLGICs) are important neurotransmitter receptors in the human central nervous system (CNS). The pLGIC family includes the γ-aminobutyric type A receptors (GABA_A_Rs), nicotinic acetylcholine receptors (nAChRs), the 5-hydroxytryptamine type 3 receptor (5-HT_3_R) and, of particular interest to us, glycine receptors (GlyRs). The proportionate activation of these receptors ensures a balance between neuronal excitation and inhibition (Corringer *et al.*, 2012 ▸; Lemoine *et al.*, 2012 ▸; Thompson *et al.*, 2010 ▸), and mutations that perturb the balance are associated with neurological and psychiatric disorders (Helbig *et al.*, 2008 ▸; Shiang *et al.*, 1993 ▸). The pharmacological relevance of pLGICs is well recognized, with members being targeted by anesthetics or drugs to treat anxiety as examples (Lemoine *et al.*, 2012 ▸; Olsen, 2018 ▸). The successful use of relatively few drugs against the large pLGIC family suggests future opportunities for drug discovery if an improved understanding of specific structure–activity relationships, appropriate chemical tools and tech­niques were available. However, there are inherent difficulties in targeting complex, multi-subunit membrane-bound ion channels for drug discovery. The presence of detergents can complicate compound screens, and multiple ligand-binding sites that vary depending on the conformational state of the ion channel are also problematic. To this we add the very significant complication that the overwhelming majority of human pLGICs of physiological and pharmacological relevance are heteromeric, with distinct subunit combinations that display unique biophysical and pharmacological profiles. These assemblies are unevenly distributed throughout the CNS and its periphery, and the heterogeneity provides an opportunity for the development of ligands with receptor-subtype specificity (Dutertre *et al.*, 2012 ▸; Shan *et al.*, 2012 ▸; Webb & Lynch, 2007 ▸). In large part, owing to difficulties in the recombinant protein production of heteromeric samples, structural studies are largely restricted to homomeric pLGICs, with a limited capacity to characterize the details of selectivity that can guide the development of selective chemical probes necessary to support fundamental studies or drug discovery. There have been modeling exercises (Bergmann *et al.*, 2013 ▸; Richter *et al.*, 2012 ▸) and very recently highly significant progress with studies of the heteromeric human α4β2 nAChR (Morales-Perez *et al.*, 2016 ▸; Walsh *et al.*, 2018 ▸) and αβγ GABA_A_ receptors (Laverty *et al.*, 2019 ▸; Masiulis *et al.*, 2019 ▸; Phulera *et al.*, 2018 ▸; Zhu *et al.*, 2018 ▸).

**Figure 1:**
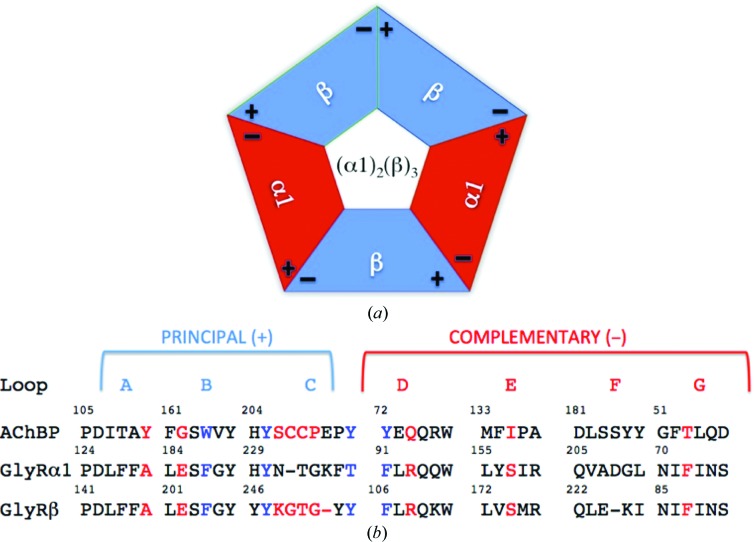
A schematic of a heteropentameric GlyR. The stoichiometry is (α1)_2_(β)_3_, with the α1 subunit in red and the β subunit in cyan. Plus and minus symbols indicate the positions of the principal and complementary sides of the binding site, respectively. In this arrangement there are three types of binding site: two α1(+)/β(−), two α1(−)/β(+) and one β(+)/β(−). (*b*) Comparison of the loop segments that create the orthosteric ligand-binding sites in *Ac*AChBP, human GlyR-α1 and GlyR-β. The residues colored red indicate where amino-acid substitutions have been carried out to create GBP. The four residues colored blue contribute to the binding site but have not been changed owing to structural conservation.

Our interest is the GlyR subtype, a particularly appealing target for the development of novel muscle relaxants and the treatment of neuropathic pain (Burgos *et al.*, 2016 ▸; Imlach, 2017 ▸; Lynch, 2009 ▸; Lynch *et al.*, 2017 ▸). The prevalent forms of human GlyR are α1β heteropentamers with 2:3 or 3:2 stoichi­ometry (Durisic *et al.*, 2012 ▸; Grudzinska *et al.*, 2005 ▸; Yang *et al.*, 2012 ▸). Several lines of evidence support the existence of mammalian glycine receptors with the 2α1:3β stoichiometry. Firstly, mutagenesis experiments implicate residues in the β subunit in binding glycine to α1β receptors expressed in *Xenopus* oocytes (Grudzinska *et al.*, 2005 ▸). Secondly, experiments with concatenated α1-β tandem constructs demonstrated that functional receptors were only expressed with the additional inclusion of the β subunit, but not the α1 subunit, implicating 2α1:3β. Thirdly, atomic force microscopy with epitope-tagged α1 and β subunits expressed in HEK293 cells indicated a 2α1:3β stoichiometry (Yang *et al.*, 2012 ▸). Mutations affecting key residues in the orthosteric agonist site at the α1(−)/β(+) interface affect the potency of both activation by glycine and inhibition by strychnine (Grudzinska *et al.*, 2005 ▸). We therefore set out to generate a high-fidelity surrogate of this α1(−)/β(+) orthosteric binding site using the 2α1:3β stoich­iometry [Fig. 1 ▸(*a*)] by exploiting protein-engineering methods and the thermal stability of acetylcholine-binding protein from *Aplysia californica* (*Ac*AChBP). Acetylcholine-binding protein is a highly conserved ortholog of the pLGIC extracellular ligand-binding domain (ECD) with properties similar to nAChR (Lemoine *et al.*, 2012 ▸; Rucktooa *et al.*, 2012 ▸; Sauguet *et al.*, 2015 ▸; Shahsavar *et al.*, 2016 ▸; Sixma & Smit, 2003 ▸). Studies on *Ac*AChBP and the *Lymnaea stagnalis* protein (*Ls*AChBP) have defined the selective recognition of ligands and provided surrogates for the excitatory nAChR and 5-HT_3_R ECDs (Kesters *et al.*, 2013 ▸; Price *et al.*, 2016 ▸). We outline comparative informatics that guided decision making and the characterization of the resulting proteins as we, in stepwise fashion, converted *Ac*AChBP to a glycine-binding protein (GBP) displaying an orthosteric site with the structural features of heteromeric human α1(−)/β(+) GlyR. Crystallo­graphic and cryo-EM structures of homomeric human GlyR-α3 (Huang *et al.*, 2015 ▸; Huang, Chen *et al.*, 2017 ▸; Huang, Shaffer *et al.*, 2017 ▸) and zebrafish GlyR-α1 (Du *et al.*, 2015 ▸) provide templates that validate our approach.

## 2. Materials and methods   

### 2.1. Site-directed mutagenesis and protein production   

The amino-acid sequences corresponding to *Ac*AChBP (Q8WSF8) and human GlyR-α1 (P23415) and GlyR-β (P48167) were retrieved from UniProt (http://www.uniprot.org/). Our numbering scheme correlates with the full-length sequences in these entries. A series of models were prepared using *Phyre* (Kelley *et al.*, 2015 ▸). Sequences were aligned with *Clustal Omega* (Sievers & Higgins, 2014 ▸), and the structure of *Ac*AChBP (for example PDB entry 2xys; Brams *et al.*, 2011 ▸) and homology models were inspected, and mutations were modeled in *Coot* (Emsley & Cowtan, 2004 ▸) to inform the design of substitutions. A stepwise approach was adopted, leading to assessments of which substitutions were important and tolerated, *i.e.* produced soluble, stable protein that was able to bind known ligands, taking into consideration ligand selectivity compared with the wild type (WT). The DNA encoding *Ac*AChBP, together with several other constructs, was purchased from GenScript. Site-directed mutagenesis was carried out and altered genes were ligated into the pFastBac system for secretion using the baculovirus/*Sf*9 system. Protein preparation followed published methods (see, for example, Hansen *et al.*, 2004 ▸) and included the use of affinity and size-exclusion chromatography.

### 2.2. Thermostability and ligand binding   

Fluorescence-based screening by differential scanning fluorimetry (DSF; see, for example, Eadsforth *et al.*, 2012 ▸) was used to determine the melting temperature (*T*_m_) values. An Mx3005P RT PCR system (Stratagene) was used to monitor protein unfolding by the increase in fluorescence of SYPRO Orange dye (Invitrogen). Assays were carried out in 40 µl volumes with proteins at around 10 µ*M* in 50 m*M* Tris–HCl, 250 m*M* NaCl pH 7.5 in 96-well RT PCR plates (ABgene). To investigate the influence of the chemical probe strychnine, 1 µl of strychnine dissolved in DMSO or buffer and then diluted with buffer was incubated with the protein solutions for 5 min prior to 71 cycles of 1°C temperature increments starting at 25°C. After each 1°C increase the sample was excited at 492 nm and fluorescence emission was recorded at 610 nm. The melting temperatures were plotted against a reference control sample of DMSO only. The strychnine concentration varied between 0.1 and 65 m*M*, with a requirement to limit the concentration of DMSO in the final mixture to <2.5%. Data are presented in Supplementary Table S1.

### 2.3. Isothermal titration calorimetry (ITC)   

The interaction of strychnine with *Ac*AChBP and GBP was investigated using ITC. Measurements were carried out with a MicroCal PEAQ-ITC (Malvern Panalytical) at 25°C. The protein solutions (10 µ*M* *Ac*AChBP, 40 µ*M* GBP) were prepared by dialysis against buffer (50 m*M* Tris–HCl pH 7.5, 250 m*M* NaCl) at 4°C overnight. Strychnine solutions (concentrations of 100 and 500 µ*M*) were prepared in the same buffer. For the experiments, the initial injection of one 0.4 µl aliquot was followed by 17 × 2 µl injections at 3 min intervals. In each case the injection needle acted as a paddle, stirring the cell contents at 750 rev min^−1^, and the reference was set at 10 µcal s^−1^. Data were analyzed using the software supplied by the manufacturer to calculate *K*_d_, Δ*H*, −*T*Δ*S*, Δ*G* and *N*, which were derived from a one-binding-site model. Control measurements, buffer into buffer, strychnine into buffer and buffer into protein solutions, were used to determine an appropriate offset adjustment. Examples of the data, averaged parameters and standard errors derived from three titrations are presented in Supplementary Fig. S1.

### 2.4. Tryptophan fluorescence-quenching assay   

Measurements were recorded using a PerkinElmer LS-55 spectrophotometer with the detector sensitivity set to 750 V. Stock solutions of 10 µg ml^−1^ GBP and *Ac*AChBP were prepared, along with two strychnine stock solutions of 100 µ*M* and 1 m*M* in the same buffer as used for the ITC experiments. The protein samples (2 ml) were excited at a wavelength of 280 nm, and emission was recorded between 300 and 400 nm. For GBP, aliquots of 20 µl of the 1 m*M* strychnine stock were used, followed by mixing. For AChBP, additions of 2 µl of the 100 µ*M* strychnine stock were made, followed by mixing. Experiments were carried out in triplicate and the percentage change in fluorescence was calculated. Data were analyzed using *Microsoft Excel* and *GraphPad Prism* 7. Examples of the data, parameters and standard errors derived from three titrations are presented in Supplementary Fig. S2.

### 2.5. Crystallographic analyses   

Each protein sample (4 mg ml^−1^ in 50 m*M* Tris–HCl pH 7.5, 250 m*M* NaCl) was incubated with the appropriate ligand for 1 h before setting up crystallization trials using sitting-drop vapor diffusion with standard sparse-matrix screens. Initial conditions were identified and then optimized for each sample (Supplementary Table S2). Ultimately, this led to six distinct crystal forms. Crystals were harvested using a nylon loop, cryoprotected with reservoir solution adjusted to contain 30% ethane-1,2-diol or 30% glycerol and then flash-frozen in liquid N_2_. Diffraction data were recorded in-house, using beamline I04-1 at Diamond Light Source or beamline ID23-1 at the European Synchrotron Radiation Facility. Images were indexed and integrated using *XDS* (Kabsch, 2010 ▸). The data were scaled using *AIMLESS* (Evans & Murshudov, 2013 ▸) from the *CCP*4 suite (Winn *et al.*, 2011 ▸) and the structures were solved by molecular replacement with *Phaser* (McCoy *et al.*, 2007 ▸). The initial model for molecular-replacement calculations was the wild-type structure (PDB entry 2xys). Multiple rounds of automated restrained refinement in *REFMAC*5 (Murshudov *et al.*, 2011 ▸) combined with electron-density and difference density map inspection and interpretation using *Coot* (Emsley & Cowtan, 2004 ▸) were carried out. Asn91 is glycosylated and *N*-acetyl-d-glucosamine was modeled onto several subunits. Whilst inspecting the different maps it was clear that additional ligands present in the crystallization mixture were ordered in the structures. These were assigned and refined satisfactorily as chloride, citrate, ethane-1,2-diol, isopropyl alcohol or phosphate. Water molecules and side-chain conformers were included in the models as appropriate. The asymmetric units of the different crystal forms contained either five, ten or 15 subunits, and strict noncrystallographic symmetry restraints were applied during most of the refinement and were relaxed towards the end of the process. Dictionaries of ligand restraints were assembled using *grade* (Smart *et al.*, 2014 ▸). Model geometry was assessed with *MolProbity* (Chen *et al.*, 2010 ▸) and the PDB validation tools. Figures were generated using *PyMOL* (Schrödinger). Further details, including relevant statistics, are presented in Supplementary Table S3 and Fig. S3.

## 3. Results and discussion   

### 3.1. Comparison of *Ac*AChBP and GlyR sequences to inform surrogate design   

**Figure 2:**
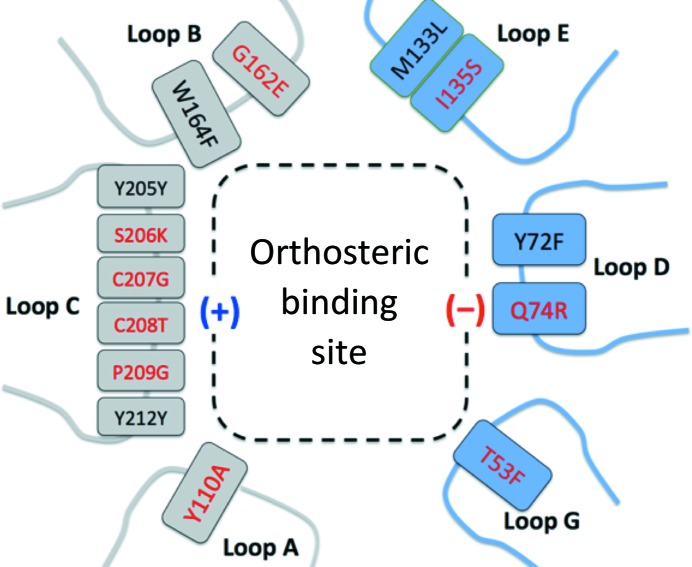
Schematic to describe the construction of and key residues in the orthosteric binding site of *Ac*AChBP and the corresponding amino acids in the human GlyR-α1(−)/β(+) heteromeric site. Substitutions in red convert *Ac*AChBP into GBP.

The alignment of the amino-acid sequences of *Ac*AChBP, human GlyR-α1 and human GlyR-β [Fig. 1 ▸(*b*)], and homology modeling together with published mutagenesis data (see, for example, Grudzinska *et al.*, 2005 ▸; Pless, Hanek *et al.*, 2011 ▸; Pless, Leung *et al.*, 2011 ▸; Yu *et al.*, 2014 ▸) on the effects of specific substitutions were used to guide the conversion of the orthosteric site of *Ac*AChBP to that of a human GlyR-α1(−)/β(+) heteromer. The orthosteric site is constructed at the subunit–subunit interface by seven loop segments. Three of these loops (labeled A–C) form the (+) or principal side of the site and four (labeled D–G) form the (−) or complementary side [Fig. 1 ▸(*b*)]. Loop F was judged to be sufficiently distant from the orthosteric binding site to be ignored. Residues with side chains directed into the orthosteric site were marked for attention (Fig. 2 ▸, Table 1 ▸). In stepwise fashion, we generated baculovirus expression systems encoding genes for WT and altered versions of *Ac*AChBP, purified and characterized the recombinant proteins to understand the consequences of alterations in and around the orthosteric site. DSF allowed us to measure the changes in stability (*T*_m_) as a consequence of amino-acid substitutions and ligand binding (Supplementary Table S1). Tryptophan fluorescence provided data relating to binding affinity, and crystallographic analyses of eight ligand complexes provided structural data (Supplementary Table S3, Figs. S2 and S3). Three well characterized pLGIC modulators, nicotine, tropisetron and strychnine, were used as chemical probes to provide control data.

### 3.2. Characterization of variants I and II   

The *Ac*AChBP–nicotine complex crystal structure provided a check of the orthosteric binding site and direct comparison with the *Ls*AChBP complex (Celie *et al.*, 2004 ▸), and confirmed that the binding sites and protein–ligand interactions are highly conserved. The orthosteric site is a narrow hydrophobic cavity dominated by five aromatic residues on one side, a disulfide bond and four aliphatic residues on the other. Of 20 residues that contribute to this site (Table 1 ▸), 17 are conserved between *Ac*AChBP and *Ls*AChBP, with only three differences of note: Val125 in *Ac*AChBP changes to arginine, Thr53 to lysine and Gln55 to isoleucine. The Thr53/Gln55 combination contributes to the positioning of Tyr72, which interacts directly with ligands. In *Ls*AChBP, the aliphatic parts of the lysine and the isoleucine side chains help to position a tryptophan, which occupies the place of Tyr72 in *Ac*AChBP. In *Ac*AChBP, the side chain of Val125 contributes to a hydrophobic surface of the orthosteric site and also serves to position the side chain of Arg96, which participates in an inter-subunit salt bridge with Glu170. In *Ls*AChBP, the glutamate is conserved and the equivalent residues to Arg96 and Val125 are serine and arginine, respectively. The smaller serine side chain provides space for the arginine to occupy the same position to also form a salt bridge with the conserved glutamate, whilst the aliphatic component of the arginine essentially mimics the contributions of Val125 to the binding site.

Variant I incorporated five changes: T53F, Q74R, Y110A, I135S and W164F. The key observation from the structure of the variant I strychnine complex concerned the Y110A, W164F and I135S substitutions. Trp164 NE1 donates a hydrogen bond to the carbonyl of Ile135, thus linking two β-strands from different subunits. Removal of the stabilizing interaction is likely to contribute to Δ*T*_m_ of this variant (−40°C) compared with the wild-type protein. We also note a biphasic melting curve that may represent first dissociation of the pentamer followed by unfolding of the subunit. The electron density of the phenylalanine (Phe164) was poorly ordered, perhaps as the reduction in the side-chain size of an adjacent residue (Y110A) opened up one side of the binding site, allowing a greater degree of conformational freedom. This variant nevertheless retained the ability to bind strychnine, as revealed in the complex crystal structure and by a Δ*T*_m_ of +20°C. The structure also indicted that the Y110A substitution created space to accommodate a G162E substitution (see later). Thr108 abuts Trp164 on adjacent β-strands in *Ac*AChBP, and we reasoned that Trp164 could be retained since the residue equivalent to the adjacent Thr108 is Phe144 in human GlyR-β and the six-membered ring of the indole would replicate the Phe144/Phe204 combination in the human system. The retention of tryptophan also preserved the ability to exploit fluorescence measurements for binding studies.

Variant II therefore reverted back to Trp164, but with the inclusion of a G162E substitution. The residues now changed (Thr53, Gln74, Tyr110, Ile135 and Gly162) correspond to Phe72, Arg93, Ala146, Ser157 and Glu202 in the human GlyR-α1(−)/β(+) orthosteric site [Figs. 1 ▸(*b*) and 2 ▸]. The important aromatic residues Tyr72 (Phe91), Trp164 (Phe204), Tyr205 (Tyr247) and Tyr212 (Tyr253) are well conserved in the two systems [Figs. 1 ▸(*b*) and 2 ▸]. The *T*_m_ of 80°C for variant II is an increase of 25°C compared with variant I and is only 10°C lower compared with the WT protein. The incorporation of the inter-strand hydrogen bond between Trp164 and Ile135 is likely to support this recovery of thermal stability. Strychnine binding to variant II resulted in a Δ*T*_m_ of +5°C.

**Figure 3:**
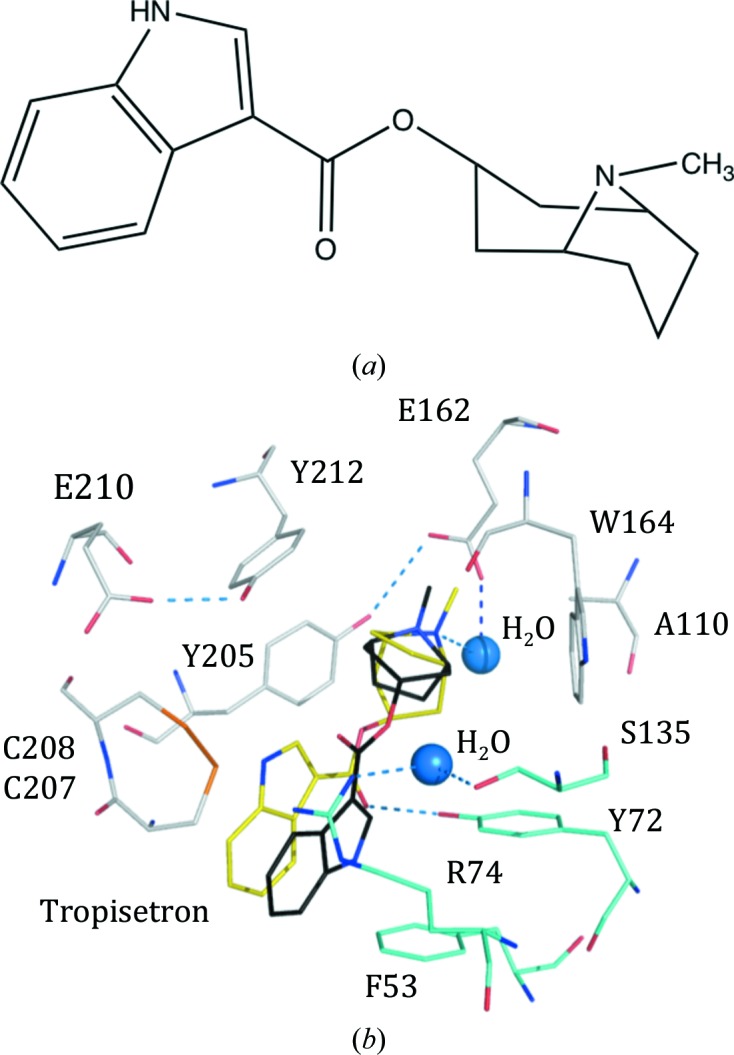
Tropisetron adopts two poses in the orthosteric site of variant II. (*a*) The chemical structure of tropisetron. (*b*) The interacting residues of variant II are shown with C positions colored white for the principal side and cyan for the complementary side, with one tropisetron pose (yellow C positions). Two water molecules discussed in the text are depicted as blue spheres; O and N positions are red and blue, respectively. Selected hydrogen-bonding interactions are shown as blue dashed lines. The second pose, which is common with that adopted in WT *Ac*AChBP (PDB entry 2wnc), is shown with black C atoms.

Crystal structures of variant II with HEPES, tropisetron and strychnine revealed that the G162E substitution was accommodated with the structure essentially unperturbed compared with variant I, although now incorporating two charged residues (Arg74 and Glu162) to polarize the binding site such that the principal side is negatively charged and the complementary side is positive. The complex structure with HEPES showed this crystallization buffer component binding in two orientations in the orthosteric site in a similar fashion to that reported for *Ls*AChBP (Celie *et al.*, 2004 ▸). The variant II complex showed tropisetron [Fig. 3 ▸(*a*)] to be present in two of the ten orthosteric sites in the asymmetric unit, with the other sites being occupied by the cryoprotectant ethane-1,2-diol and the N-terminal histidine tails of symmetry-related molecules. Although the tropisetron occupies the same space, our interpretation of the electron density is that this modulator of GlyR (Yang *et al.*, 2007 ▸) displays two poses [Supplementary Figs. S3(*c*) and S3(*d*)]. One pose is similar to that observed in the WT *Ac*AChBP (Hibbs *et al.*, 2009 ▸) complex, whilst the other is rotated approximately 180° (Fig. 3 ▸). When bound to the WT protein, the tropane-bridged piperidine binds in the same position as the pyrrolidine moiety of nicotine, forming van der Waals interactions with the side chains of Tyr72 from one subunit and Tyr205, Tyr212 and Trp164 from the other subunit. The quaternary amine N1 donates a hydrogen bond to the carbonyl of Trp164 and the methyl substituent forms van der Waals interactions with Tyr110. A solvent-mediated hydrogen-bonding network links the tropisetron carbonyl to the carbonyl of Val165 and Tyr212 hydroxyl group of one subunit and the carbonyl groups of Ile106 and Met133 on the partner subunit. The ether/carbonyl link between the tropane and indole groups forms van der Waals contacts to the Cys207–Cys208 disulfide part of loop C on the (+) side and the side chain of Ile135 on the (−) side. The indole group is positioned with van der Waals contacts to Cys207 on one side and to the side chains of Tyr72, Gln74 and Met133 on another subunit. The indole N10 forms a hydrogen bond to a water molecule, which in turn interacts with Thr53 and Asp181 and other solvent molecules that form a network of hydrogen bonds in and around the binding site. The second pose is influenced by the Y110A and G162E substitutions, which allow a solvent-bridged interaction between the glutamate and tropisetron N1. van der Waals interactions between the tropane and aromatic residues are maintained in both poses with minor adjustments of side chains. The T53F substitution and the placement of the aromatic group help to place the arginine from the Q74R substitution to participate in a cation–π stacking arrangement of the guanidinyl moiety and the indole system, pushing tropisetron over towards the disulfide linkage on loop C. A solvent-mediated link between Ser135 and Arg74 may also contribute to the placement of the guanidinyl moiety. The indole N10 is directed out towards bulk solvent, whilst the carbonyl group accepts a hydrogen bond donated from the side chain of Tyr72.

Of note is the observation that tropisetron can adopt two poses in the same binding site. It is not unusual to observe a statistical disorder in which two orientations of a ligand are present in the population of molecules in a crystal (see, for example, Khalaf *et al.*, 2014 ▸). The possibility exists that here also tropisetron adopts more than one orientation in the binding site, in effect a mixed population, but the electron-density maps suggest a dominant pose in each of the two binding sites that are occupied [Supplementary Figs. S3(*c*) and S3(*d*)]. This observation matches well with previous work on tropisetron and derivatives interacting with the 5-HT_3_R that indicate that different binding orientations are possible (Ruepp *et al.*, 2017 ▸).

The *K*_d_ values for the binding of nicotine and tropisetron to *Ac*AChBP are 250 and 480 n*M* by monitoring intrinsic tryptophan fluorescence quenching with stopped-flow spectrofluorimetry (Hansen *et al.*, 2005 ▸). Comparable values were obtained with our tryptophan fluorescence measurements: 245 (±20) and 275 (±15) n*M*. This validated assay was applied to investigate how the substitutions might influence ligand affinity. Neither variant I nor variant II appeared to be able to bind nicotine. The combined Y110A and I135S substitutions may open up the binding site such that nicotine can longer bind in an optimal fashion. However, we were unable to co-crystallize these variants with glycine, neither did the fluorescence assay register any glycine binding.

### 3.3. Variant III is a glycine-binding protein   

Variants I and II presented structural features consistent with site-directed mutagenesis and electrophysiological data that suggest interacting roles for specific residues (see, for example, Yu *et al.*, 2014 ▸). However, our structures also emphasized that accurately replicating the α(−)/β(+) heteromeric site required changes to the β(+) side loop C, where the major differences between α-form and β-form sequences occur (Figs. 1 ▸ and 2 ▸). Single-site substitutions were not obvious and we judged it necessary to make a major change, with four substitutions being incorporated (S206K, C207G, C208T and P209G). These substitutions had the potential to release the conformational restraint of the Cys207–Cys208 disulfide and, with two glycine residues now included, to increase the conformational mobility of the loop. Variant III was produced in recombinant form and characterized.

The substitutions did not have an adverse effect on the stability of the protein, with *T*_m_ values of 80 and 81°C noted for variants II and III, respectively (Supplementary Table S1). The binding of strychnine to variant III led to a small increase, +3°C, in Δ*T*_m_, and when glycine was tested a Δ*T*_m_ of +2°C was observed. These changes are small and are unlikely to be significant. Attempts to observe an association between variant III and glycine using the fluorescence assay, ITC and biolayer interferometry failed to show any binding. This may be a consequence of testing a compound with such a low mass (about 75 Da). However, variant III co-crystallized with glycine and the ligand occupies four of the five orthosteric binding sites in the asymmetric unit. With this proof of binding we named variant III glycine-binding protein (GBP). The molecular packing in the crystal lattice of the GBP–glycine complex places a histidine from the affinity tag in the other site. The structure of wild-type *Ac*AChBP in complex with strychnine, the archetypal GlyR antagonist, reported here as a control (PDB entry 2xys; Brams *et al.*, 2011 ▸) provided a comparison for the GBP–strychnine complex, which we also crystallized. During our study, crystal structures of homomeric human GlyR-α3 complexes with glycine (PDB entry 5tin) and strychnine (PDB entry 5cfb) became available (Huang, Chen *et al.*, 2017 ▸; Huang, Shaffer *et al.*, 2017 ▸), allowing direct comparisons.

**Figure 4:**
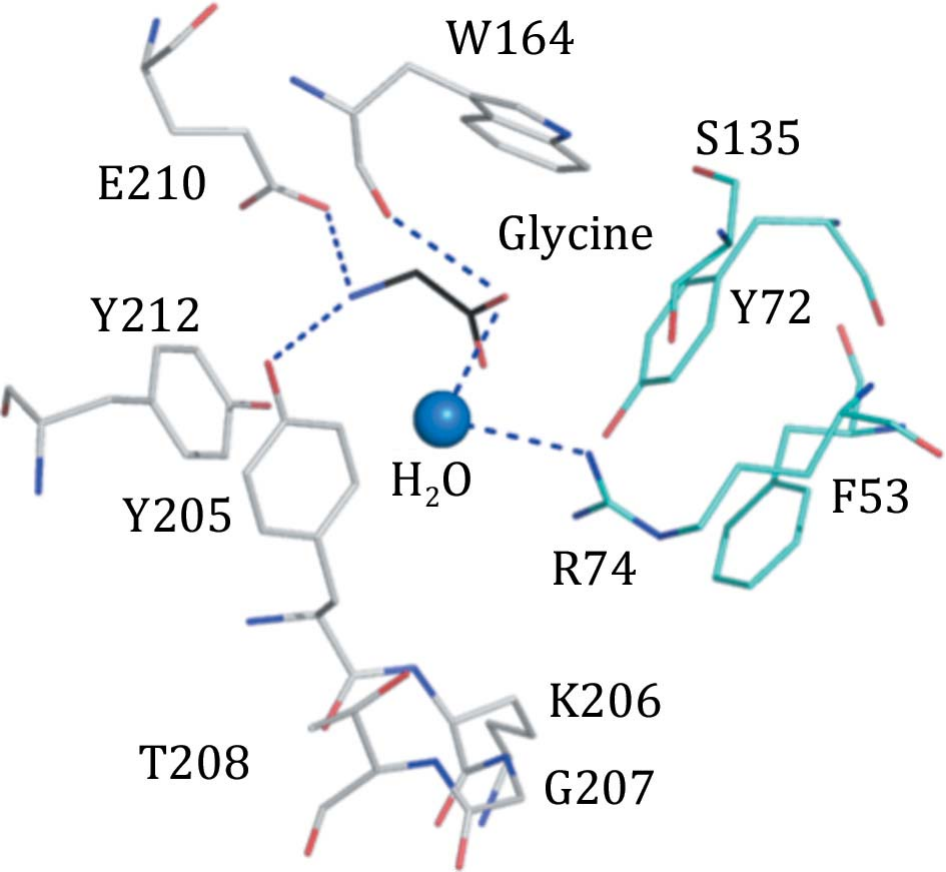
Glycine in an orthosteric site of GBP. A similar color scheme as shown in Fig. 3 ▸ is used, with glycine C positions in black.

The orthosteric site in the GBP–glycine structure is highly conserved with that of the WT GlyR homomer structures and variants I and II, with the notable exception of loop C, which now adopts a configuration that allows two tyrosine residues (Tyr205 and Tyr212) to contribute to the binding site (Fig. 4 ▸). One edge of the Tyr205 side chain helps to form one side of the binding site, with the hydroxyl group placed to donate a hydrogen bond to the carbonyl of Tyr166 (not shown), thereby linking two segments, and to accept a hydrogen bond from the glycine ligand. Tyr212 is aligned with Tyr205, creating a π-electron-rich region to interact with the glycine amino group. Solvent-mediated interactions link the Tyr212 hydroxyl with the carboxylate of Glu162 (not shown), which in turn interacts with the glycine amino group. The glycine is tucked between and participates in van der Waals interactions with the edge of Tyr205 and the face of the Trp164 indole. The glycine carboxylate is directed towards the Arg74 guanidinyl moiety, but the distances (≥3.8 Å) are too long to represent direct hydrogen-bonding interactions and a solvent-mediated association is noted.

In the structure of the homomeric GlyR-α3 glycine complex (Huang, Shaffer *et al.*, 2017 ▸), the glycine carboxylate accepts hydrogen bonds donated by the side chains of Arg65, Ser129 and Thr204, the latter on loop C. The amino group of the ligand makes a solvent-mediated interaction with Glu157 and a direct hydrogen bond to the carbonyl of Phe159. The glycine participates in van der Waals interactions with the side chains of Phe63 and Phe159, whilst the amino group occupies a π-electron-rich area between Phe159 and Phe207. A water molecule bridges this amino group to the carbonyl of Ser158 and the carboxylate of Glu157 with three hydrogen bonds. The major differences between the two structures and the key to forming a heteromeric site reside in loop C, the part of the binding site that distinguishes the α- and β-forms of the receptor. In GlyR-α3 the loop segment comprising residues 199–207 is in a closed conformation, whilst the equivalent residues 202–211 in GBP form a more open structure. However, in GBP the loop conformation places the side chains of the tyrosine pair (205 and 212) directed into the binding site, whereas in GlyR-α3 only one tyrosine, Tyr202, is thus positioned, forming a hydrogen bond with the conserved acidic Glu157.

**Figure 5:**
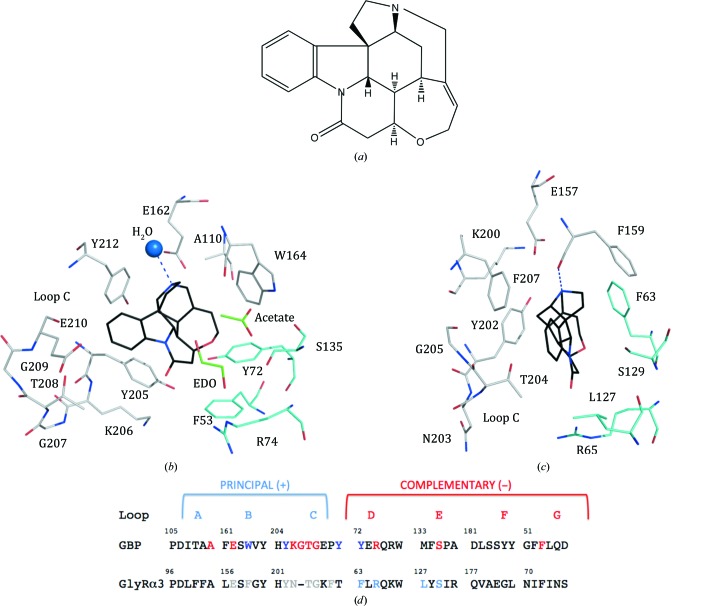
Strychnine bound to GBP. (*a*) The chemical structure of the natural product. (*b*) The key residues and orientation of strychnine bound to GBP. A similar color scheme as shown in Fig. 3 ▸ is used, with C positions of strychnine in black and C positions of acetate and ethanediol (EDO) in green. (*c*) The binding of strychnine to the human GlyR-α3 homomer from PDB entry 5cfb (Huang *et al.*, 2015 ▸); the residue numbers in the PDB entry are retained. (*d*) For comparative purposes the alignment of GBP [see Fig. 1 ▸(*b*)] with human GlyR-α3 is shown using the numbering scheme of the PDB entry. Residues shown in (*c*) are shown in gray for the principal side and in cyan for the complementary side.

The structure of the GBP–strychnine complex displays a well ordered ligand in three out of five binding sites per asymmetric unit. In two sites the density is less clear, the thermal parameters are elevated (Supplementary Table S3) and different strychnine orientations are noted. A previously published structure of the WT *Ac*AChBP–strychnine complex shows a single molecule occupying four of the five orthosteric sites in the asymmetric unit and one site with two ligands bound (Brams *et al.*, 2011 ▸). In both of these structures the molecular packing in the crystal lattice affects the conformation of loop C in one subunit and results in a more open binding site, providing room for ligands to adopt different orientations. We note also that this alkaloid displays a propensity to dimerize or aggregate at high concentrations (Reinscheid *et al.*, 2016 ▸). Our structure of the WT *Ac*AChBP–strychnine complex displays a single well ordered strychnine in all five binding sites per asymmetric unit and we confine our comparison to this binding pose (Fig. 5 ▸).

The orientation and the position of the alkaloid in both WT *Ac*AChBP structures is very similar. There is a single direct hydrogen bond between the alkaloid and the protein donated by the protonated tertiary amine to the carbonyl of Trp164. The p*K*_a_ of strychnine is approximately 8.3 (Haynes, 2015 ▸), hence the confidence that protonation has occurred. The potential hydrogen-bond acceptors on strychnine, ether and amide O atoms, are placed to interact with solvent and in so doing then form bridges to the protein. Extensive van der Waals interactions involving four tyrosine residues (110, 205, 212 and 72), Trp164, Met133, Ile135 and the Cys20–Cys208 disulfide are likely to explain the high affinity of strychnine for this binding site, with a reported *K*_i_ of 38 n*M* (Brams *et al.*, 2011 ▸) and *K*_d_ of 15 n*M* (Hansen *et al.*, 2004 ▸), the latter based on a stopped-flow spectrofluorimetry assay. The use of ITC and a radioligand saturation assay to characterize the interaction between strychnine and a recombinant homomeric GlyR-α1 system gave *K*_d_ values of 138 (±55) and 52 (±6) n*M*, respectively (Wöhri *et al.*, 2013 ▸). The application of ITC and surface plasmon resonance methods with recombinant homomeric GlyR-α3 produced *K*_d_ values of 52 (±2) and 43 (±3) n*M*, respectively (Huang *et al.*, 2015 ▸). For comparative purposes we employed ITC to characterize the binding of strychnine to WT *Ac*AChBP and GBP (Supplementary Fig. S1). The resulting *K*_d_ for the interaction with WT *Ac*AChBP is 74.1 (±22.6) n*M* and that with GBP is 28.8 (±3.2) µ*M*. Corroboration of these data was sought using a fluorescence-based assay, where *K*_d_ values of 155 (±7) n*M* and 27 (±0.5) µ*M* were determined for WT *Ac*AChBP and GBP, respectively.

Strychnine is a promiscuous ligand that is active against different receptors. Electrophysiological assays indicate that the alkaloid, although the prototypical competitive antagonist of GlyR, also displays the same activity against some nAChR subtypes, with IC_50_ values that range from 350 n*M* to around 40 µ*M* (Albuquerque *et al.*, 1998 ▸; García-Colunga & Miledi, 1999 ▸; Jensen *et al.*, 2006 ▸). The affinity of strychnine interacting with *Ac*AChBP is consistent with the activity that this ligand displays against the orthologous binding sites presented by nAChRs. The substitutions that were introduced to engineer a glycine-binding site in GBP have however reduced the affinity for strychnine significantly away from that of the wild-type template as well as from that observed with recombinant homomeric GlyR samples. The thermodynamic parameters [Supplementary Fig. S1(*e*)] indicate that whilst the enthalpic contribution to strychnine binding is similar for *Ac*AChBP and GBP, there is a significant penalty in the entropic contribution that explains the reduced affinity of GBP for this ligand. We speculate that this may be linked to a reduction in thermal stability of GBP relative to the wild-type protein and/or be influenced by the increased flexibility introduced into loop C. The observation does have an important implication for our use of the GBP surrogate, suggesting that care should be applied when using this system with larger ligands that might engage with residues distant from those involved directly in neurotransmitter binding.

The WT *Ac*AChBP, GlyR-α3 homomer and GBP complexes display different orientations of strychnine in the binding site that are directly linked to the substitutions that have been incorporated to produce GBP (Fig. 5 ▸). An overlay of the protein structures (data not shown) places the tertiary amines within 2 Å of each other, but the ligands adopt different poses, essentially pivoting around this N atom. In the GBP–strychnine complex the tertiary amine is protonated and donates a hydrogen bond to a highly ordered water that forms hydrogen bonds to the carbonyl groups of Ser163 and Tyr166 and contributes to a solvent network that burrows into the protein fold (data not shown). The Y110A substitution removes the possibility of van der Waals interactions between the aromatic side chain and any ligand. The G162E change directs a polar side chain into the bindng site and this forces the strychnine to adopt a different orientation. On the other side of the binding site, the Q74R substitution serves to place a polar group further into the cleft. This would clash with the ligand orientation noted in the wild-type *Ac*AChBP complex and thus works in concert with the presence of Ala110 and Glu162 to position the ligand. The I135S and T53F changes allow Tyr72 to adopt a different rotamer conformation: in the former by providing space and in the latter with stabilizing van der Waals interactions. The position of Phe53 also serves to stabilize the side-chain position of Arg74. The change in orientation of strychnine results in the amide carbonyl O atom rotating by about 90° and relocating by almost 8 Å directed towards Met133 and with the indole system placed to interact with loop C. Here also there are significant changes to the protein structure. The side chain of Tyr205 adopts a different rotamer, participates in van der Waals interactions with strychnine and now occupies the space that is filled by the Cys207–Cys208 disulfide bond in WT *Ac*AChBP, in essence forming a lid over the binding site. Tyr212 also displays a different rotamer, partially filling the space vacated by Tyr205, and this allows the strychnine indole moiety to bind under loop C.

## 4. Conclusions   

We sought to investigate the orthosteric binding site of the heteromeric α1(−)/β(+) form of human GlyR but circumventing the experimental difficulties of working with a multi-subunit membrane-bound protein. Based on existing sequence, structural and functional data, we considered which amino acids of the homolog *Ac*AChBP might be substituted, allowing us to create a convenient surrogate system. In stages, we modified *Ac*AChBP and characterized variant proteins to interrogate the binding site. Ultimately, the highly stable *Ac*AChBP framework has allowed us to introduce nine amino-acid substitutions that have resulted in a stable surrogate for a heteromeric neurotransmitter site that binds glycine. This system could be exploited in early-stage drug discovery by use as a target for the screening of chemical libraries, for structural elucidation of receptor–ligand interactions, and for biophys­ical characterization of kinetic and thermodynamic parameters relating to ligand binding.

## Supplementary Material

PDB reference: acetylcholine-binding protein, wild type, strychnine complex, 5o8t

PDB reference: wild type, nicotine complex, 5o87

PDB reference: variant I, strychnine complex, 5oa0

PDB reference: variant II, HEPES complex, 5oad

PDB reference: variant II, tropisetron complex, 5oaj

PDB reference: variant II, strychnine complex, 5oal

PDB reference: variant III, glycine complex, 5oan

PDB reference: variant III, strychnine complex, 5obg

Supplementary Tables and Figures. DOI: 10.1107/S205225251901114X/jt5037sup1.pdf

## Figures and Tables

**Table:** Residues in bold were substituted with the human equivalents to create glycine-binding protein (GBP).

*Ac*AChBP			GlyR-β(+)	
Residue	Loop	Role	Residue	Comment
**Tyr110**	A	Aromatic lining of the site, with hydroxyl contribution	**Ala146**	Reduction in size, makes space for Glu202
**Gly162**	B	Adjacent to Tyr110	**Glu202**	Increase in size and introduces negative charge
Ser163	B	Hydroxyl forms a hydrogen bond to the Tyr166 amide to hold Trp164 and Val165 in place	Ser203	Strictly conserved
Trp164	B	Aromatic contribution to site, inter-subunit hydrogen bond to Ile135 carbonyl	Phe204	Conserved aromatic with slight reduction in bulk, no hydrogen bond
Val165	B	Hydrophobic contribution	Gly205	Reduction in size
Tyr205, Tyr212	C	Tyrosine pair contributes aromatic lining and hydroxyls to site	Tyr247, Tyr253	Strictly conserved
**Ser206**, **Cys207**, **Cys208**, **Pro209**	C	Disulfide contributes hydrophobic lining to site and restrains the loop conformation	**Lys248**, **Gly249**, **Thr250**, **Gly251**	Changes likely to give more conformational freedom to loop C

**Table:** 

*Ac*AChBP			GlyR-α1(−)	
Residue	Loop	Role	Residue	Comment
Tyr72	D	Aromatic contribution	Phe91	Conserved
**Gln74**	D	Abuts Ile135, Met133	**Arg93**	Introduction of bulk and positive charge
Ile123, Ala124, Val125	E	Hydrophobic contributions from Val125 and Ala124 C^α^; Ile123 carbonyl directed into site	Leu145, Leu146, Arg147	Conserved Ile/Leu but increase in size for Leu146 and Arg147; aliphatic part of Arg147 side chain lines site
Met133	E	Hydrophobic lining to site, inter-subunit van der Waals interactions with loop C disulfide	Leu155	Conserved
**Ile135**	E	Hydrophobic lining	**Ser157**	Reduction in size allows space for Arg93, addition of a polar group in site
**Thr53**	G	Abuts Tyr72	**Phe72**	Increase in bulk and hydrophobicity to position Phe91
Gln55	G	Inter-subunit hydrogen bond to carbonyl serves to place Tyr110 and van der Waals interactions to position Tyr72 in site	Asn74	Conserved
