# Supplementary material for: Engineering a surrogate human heteromeric α/β glycine receptor orthosteric site exploiting the structural homology and stability of acetylcholine-binding protein
Source: IUCrJ. 2019 Sep 4;6(Pt 6):1014–23. doi: 10.1107/S205225251901114X (PMC6830221; doi:10.1107/S205225251901114X)
Supplement: Supplementary file 1 [file m-06-01014-sup1.pdf]

IUCrJ

Volume 6 (2019)

Supporting information for article:

Engineering a surrogate human heteromeric α/β glycine receptor orthosteric site exploiting the structural homology and stability of acetylcholine-binding protein

Alice Dawson, Paul Trumper, Juliana Oliveira de Souza, Holly Parker, Mathew J. Jones, Tim G. Hales and William N. Hunter

Table S1 Thermal stability (T_m) of wild-type and engineered proteins, and the stabilising influence of strychnine.

Protein	*Ac*ACBP wild-type	Variant-I	Variant-II	Variant-III (GBP*)
Substitutions		T53F, Q74R, Y110A, I135S, W164F	T53F, Q74R, Y110A, I135S, G162E	T53F, Q74R, Y110A, I135S, G162E, S206K, C207G, C208T, P209G
T_m (°C)	90	35, 60 (biphasic)	80	81
ΔT_m (°C) + strychnine	Not measured, exceeds machine limits	+5, +25 (biphasic)	+5	+3

GBP, glycine-binding protein

Table S2 Crystallisation conditions.

Protein	*Ac*ACBP Wild-type	*Ac*ACBP Wild-type	Variant-I	Variant-II	Variant-II	Variant-II	Variant-III (GBP*)	Variant-III (GBP*)
Ligand	Nicotine (5 mM)	Strychnine (0.5 mM)	Strychnine (0.5 mM)	HEPES (0.1 M)	Tropisetron (5 mM)	Strychnine (0.5 mM)	Glycine (100 mM)	Strychnine (0.5 mM)
Reservoir	0.45 M ammonium phosphate, 2 % glycerol 2 % IPA*	0.1 M magnesium chloride, 25% PEG* 3350	0.1 M magnesium chloride, 25% PEG* 3350	0.1 M HEPES, 25% PEG* 2000 MME*, pH 8	20% PEG* 3350, 0.2 M sodium citrate	20 % PEG* 3350, 0.2 M magnesium formate	2 M sodium formate, 0.1 M sodium acetate, pH 4.6	0.2 M magnesium acetate, 20% PEG 3350
Cryo-protectant	30% ethane-1,2-diol	30% glycerol	30% glycerol	Not required	30% ethane-1,2-diol	Not required	Not required	30% ethane-1,2-diol
Configuration	Hanging drop	Hanging drop	Hanging drop	Sitting drop	Hanging drop	Sitting drop	Sitting drop	Hanging drop

* GBP, glycine-binding protein; IPA *iso*-propyl alcohol, MME monomethyl ether, PEG polyethylene glycol.

Table S3 . Crystallographic statistics.

Structure	*Ac*ACBP nicotine complex	*Ac*ACBP strychnine complex	Variant-I strychnine complex	Variant-II HEPES complex	Variant-II tropisetron complex	Variant-II strychnine complex	Variant-III (GBP) glycine complex	Variant-III (GBP) strychnine complex
Unit cell a,b,c (Å) α,β,γ, (°)	210.61, 131.92, 130.44 90, 103.2, 90	74.46, 74.46, 186.75 90, 90, 120	74.87, 74.87, 185.42 90, 90, 120	73.17, 118.46, 123.77 90, 90, 90	100.24, 137.47, 103.67 90, 90.8, 90	130.90, 130.90, 190.13, 90, 90, 120	89.92, 100.27, 165.24 90, 90, 90	90.35, 100.04, 163.75 90, 90, 90
Space group	$C2$	$P3_2$	$P3_2$	$P2_12_12_1$	$P2_1$	$P3_2$	$P22_12_1$	$P22_12_1$
Source	In-house	Diamond I04-1	Diamond I04-1	Diamond I04-1	Diamond I04-1	ESRF ID23-1	In-house	In-house
Wavelength (Å)	1.54157	0.91739	0.91739	0.91739	0. 91739	0.97627	1.54157	1.54157
Subunits per asy. unit	10	5	5	5	10	15	5	5
Resolution range and outer shell	25.20-2.20 (2.24-2.20)	28.63-2.20 (2.24-2.20)	37.43 – 2.60 (2.72-2.60)	33.36 - 2.10 (2.20-2.10)	42.66 – 2.47 (2.51-2.47)	72.84 – 3.20 (3.29 – 3.20)	32.90 – 2.6 (2.69-2.60)	47.92 – 2.00 (2.03 – 2.00)
Other ligands	ethane-1,2-diol, phosphate, isopropyl alcohol	None identified	ethane-1,2-diol	ethane-1,2-diol	ethane-1,2-diol, citrate	None identified	Acetate, chloride	Acetate, ethane-1,2-diol
Total number reflections	1197284 (58084)	321877 (26743)	189132 (21870)	260835 (18809)	363051 (18162)	479129 (33732)	339153 (33083)	727656 (24678)
Unique reflections	176070 (8731)	58318 (4655)	35443 (4241)	62269 (4602)	97730 (4799)	59286 (4605)	46736 (4537)	100843 (4919)
Redundancy	6.8 (6.7)	5.5 (5.7)	5.3 (5.2)	4.2 (4.1)	3.7 (3.8)	8.1 (7.3)	7.3 (7.3)	7.2 (5.0)

R_{merge}[a]	0.115 (0.639)	0.135 (0.762)	0.131 (0.719)	0.125 (1.020)	0.051 (0.270)	0.212 (1.073)	0.149 (0.632)	0.147 (0.589)
Wilson *B* [Å^2]	27.2	23.8	35.2	32.8	34.3	51.7	17.9	19.0
Completeness [%]	99.9 (99.9)	99.1 (99.7)	99.0 (95.5)	98.3 (99.5)	97.2 (97.3)	98.7 (98.5)	99.3 (99.5)	100(100)
<I/σ(I)>	8.8 (3.3)	8.5 (2.3)	9.1 (2.2)	5.4 (1.2)	14.2 (4.3)	8.3 (2.3)	11.9 (3.2)	6.1 (1.9)
CC(1/2)	0.993 (0.675)	0.994 (0.552)	0.993 (0.601)	0.994 (0.529)	0.989 (0.854)	0.991 (0.691)	0.993 (0.818)	0.992 (0.657)
**Refinement**								
R_{work}/R_{free}[b] [%]	20.28 / 23.51	18.11 / 22.95	18.83 / 22.70	22.64 / 25.51	17.28 / 20.78	17.95 / 23.11	20.25 / 23.49	19.61/ 24.97
Number reflections for R_{work}/R_{free}	167123 / 8653	55431 / 2828	33722 / 1676	58928 / 3159	92964 / 4738	56374 / 2910	44310 / 2381	95620 / 5157
Protein residues	2065	1027	1036	1029	2080	3079	1052	1038
Ligand of interest	10	5	5	3	2	15	4	5
Water molecules	1801	588	113	165	574	0	250	749
RMSDs								
Bonds [Å]	0.0094	0.0092	0.010	0.0105	0.0182	0.0166	0.0098	0.0018
Angles [°]	1.45	1.38	1.42	1.51	0.913	2.05	1.41	1.95
Ramachandran plot								
Residues in favoured regions [%]	2043 [99.3]	1007 [98.9]	1003 [97.8]	996 [97.7]	2035 [99.1]	2859 [93.9]	1004 [97.9]	1005 [97.9]

allowed regions [%]	14 [0.7]	11 [1.1]	23 [2.2]	23 [2.3]	19 [0.9]	185 [6.1]	21 [2.1]	21 [2.1]
outlier regions [%]	0	0	0	0	0	0	1 [0.10]	0
Mean B-factors								
Protein atoms per subunit [Å^2]	32.3 / 34.2 / 34.6 / 34.8 / 35.0 / 38.5 / 35.8 / 35.5 / 37.1 / 38.5	34.3 / 37.3 / 40.7 / 36.2 / 32.7	45.3 / 56.9 / 58.3 / 50.2 / 46.9	42.3 / 45.7 / 41.5 / 39.9 / 43.9	37.6 / 41.0 / 38.1 / 37.8 / 35.9 / 42.7 / 47.8 / 43.0 / 42.2 / 44.8	66.1 / 83.3 / 78.2 / 74.4 / 70.3 / 73.8 / 86.7 / 73.6 / 68.1 / 67.3 / 100.4 / 91.4 / 81.3 / 75.5 / 100.8	28.3 / 27.0 / 27.4 / 30.3 / 32.4	25.5 / 29.9 / 29.2 / 26.3 / 26.2
Waters [Å^2]	42.0	34.5	38.8	37.1	36.2	-	19.9	32.8
Ligand of interest [Å^2]	33.4 / 39.1 / 35.5 / 34.5 / 38.0 / 42.6 / 35.5 / 44.6 / 43.7 / 43.3	54.6 / 64.0 / 42.0 / 52.4 / 52.5	37.5 / 88.7 / 59.4 / 42.2 / 45.4	70.9 / - / 71.8 / - / 58.8	TKT D 47.8/ TKT J 46.6	106.9 / 120.4 / 69.9 / 107.2 / 74.2 / 94.3 / 132.2 / 72.9 / 90.8 / 75.7 / 103.6 / 107.1 / 81.3 / 104.5 / 151.2	- / 48.0 / 57.4 / 51.0 / 62.5	41.2 / 31.6 / 47.0 / 26.1 / 35.6
**PDB Code**	5o87	5o8t	5oa0	5oad	5oaj	5oal	5oan	5obg

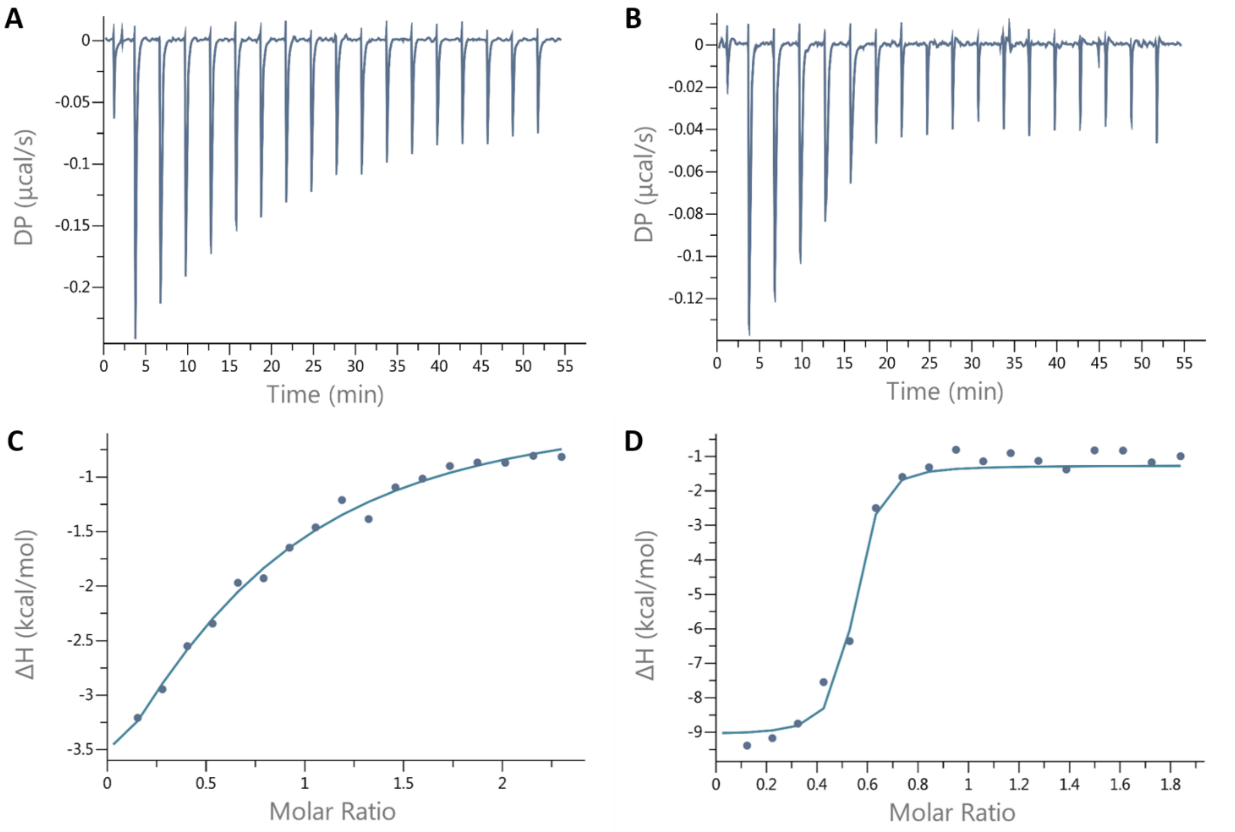

E

Protein	K_d	N (sites)	ΔG (kcal/mol)	ΔH (kcal/mol)	-TΔS (kcal/mol)
GBP	28.8 ± 3.2 µM	0.6 ± 0.0	-6.2 ± 0.1	-7.4 ± 1.0	1.3 ± 1.1
AChBP	74.1 ± 22.6 nM	0.5 ± 0.0	-9.8 ± 0.2	-7.9 ± 0.4	-1.9 ± 0.5

Figure S1 Isothermal titration calorimetry data for strychnine binding to GBP and *Ac*AChBP. An example isotherm for a single titration for GBP (A) and *Ac*AChBP (B). The corresponding plots of ΔH against molar ratio are (C) and (D) respectively. (E) Calculated parameters with standard errors from three experiments.

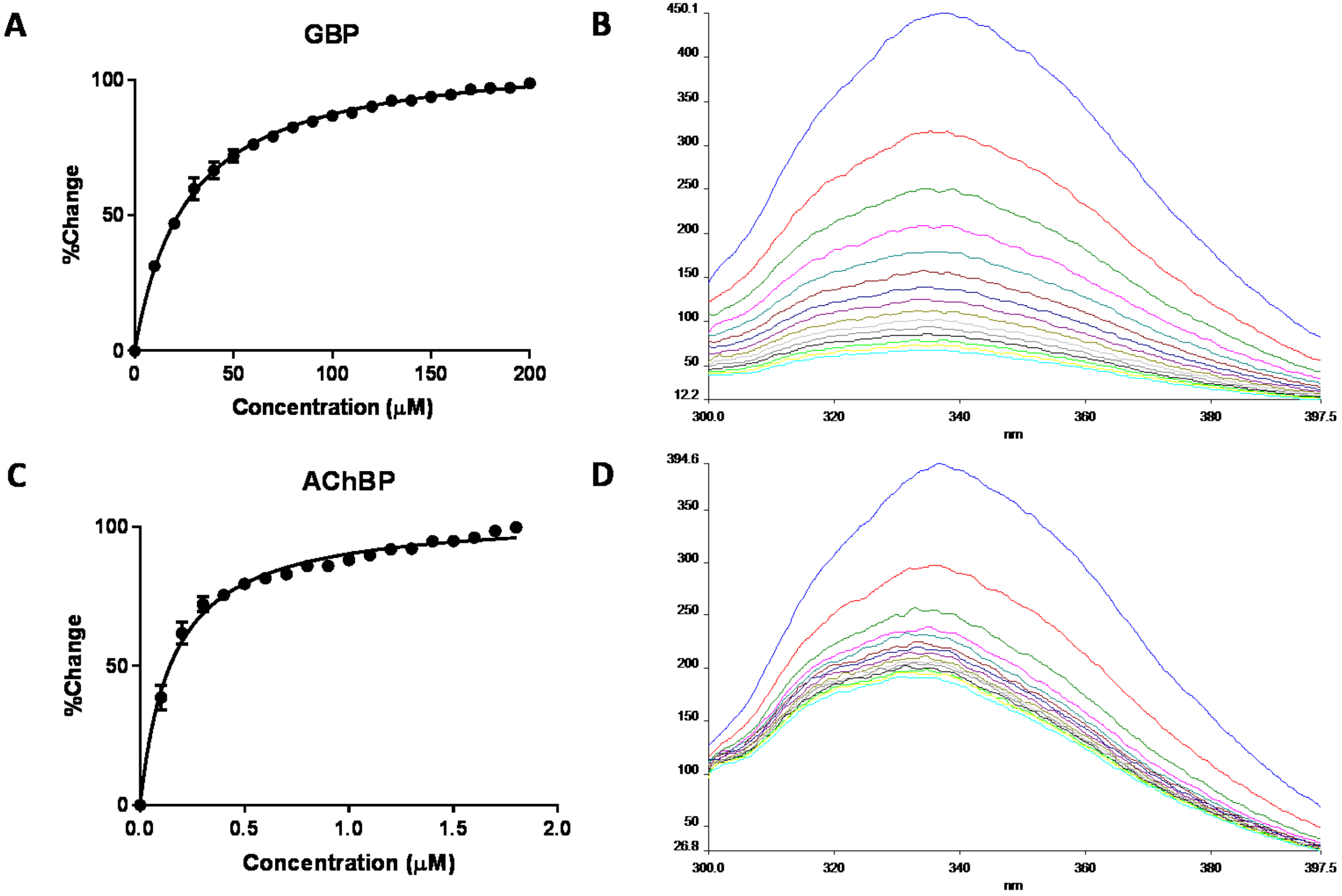

Figure S2 Tryptophan fluorescence data for strychnine binding to GBP and AChBP. (A) plot of the mean percentage change in fluorescence against concentration for GBP with standard error bars given (n=3), leading to a K_d of 26.6 ± 0.5 μM. The absorbance was recorded at a constant wavelength. (B) Example trace obtained in a titration starting with no ligand (blue) followed by incremental additions. (C) The plot for the titration with AChBP, giving a K_d of 155 ± 7 nM and (D) an example titration starting at no ligand (blue) followed by incremental additions.

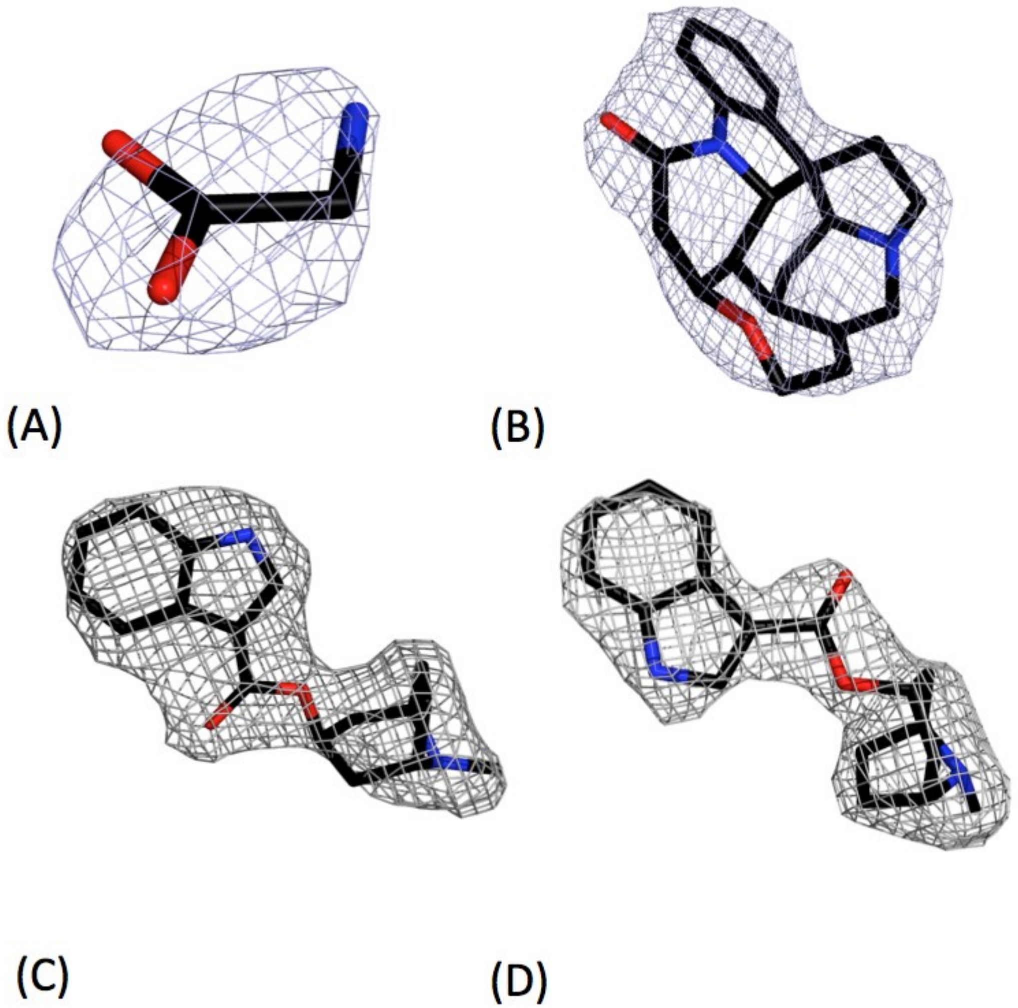

Figure S3 Representative omit difference density maps for ligands used in this study. The maps are depicted in a chicken wire effect, at the 3 r.m.s.d. level with ligands shown in stick mode with atomic positions colored black C, red O, blue N. (A) Glycine in complex with GBP (B) strychnine in complex with GBP (C) tropisetron in complex with variant-II in one orientation, (D) the alternative orientation.
